# Stercoral perforation proximal to the stapled anastomosis after low anterior resection with an intraluminal device

**DOI:** 10.1007/s00384-017-2924-3

**Published:** 2017-10-23

**Authors:** J. B. van Praagh, I. S. Bakker, K. Havenga

**Affiliations:** 1 Department of Surgery, University Medical Center Groningen, University of Groningen, Groningen, The Netherlands; 2 Treant Zorggroep, Department of Surgery, Emmen, The Netherlands

**Keywords:** Perforation, C-seal, Dysmotility, Intraluminal device

## Abstract

Stercoral perforation of the colon is a rare phenomenon and a potential life-threatening condition requiring acute intervention. A little more than 200 cases have been described to date. The mechanism is not completely understood. In this short communication, we present three patients with a colon perforation proximal to the anastomosis, similar to a stercoral perforation, following colorectal cancer resection with application of an intraluminal device, the C-seal.

## Introduction

**Fig. 1:**
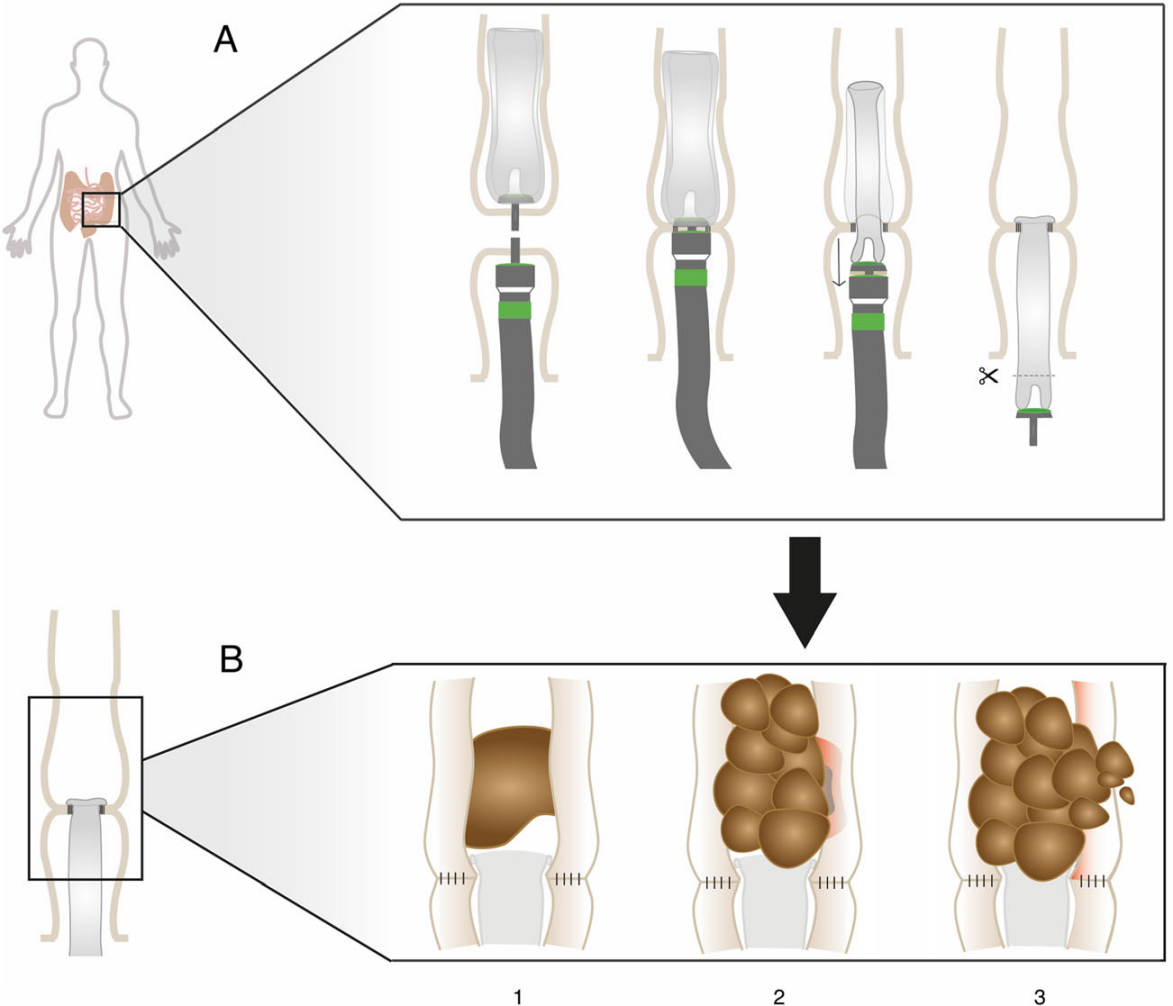
C-seal application (A) and mechanism of stercoral perforation proximal to a C-seal (B), where luminal content is found above the C-seal (1), subsequently accumulate and harden causing pressure ulceration and necrosis in the bowel wall (2); eventually leading to a perforation due to prolonged pressure necrosis of the bowel wall (3)

Stercoral colonic perforation is a rare phenomenon and a potential life-threatening condition requiring acute intervention [1]. The cause of this acute condition, however, is not completely understood. Stercoral perforation can be seen as a complication of chronic constipation, leading to bowel distension, raised intraluminal pressure with subsequent bowel perforation [1]. Diagnostic criteria for this type of colonic perforation are a round or ovoid colonic perforation, exceeding 1 cm in diameter, on the antimesenteric site of the colon; with fecalomas present in the colon protruding through the perforated site or lying within the abdominal cavity close to the colon, causing microscopic evidence of pressure necrosis or ulcers and chronic inflammation around the perforation [2]. In a few patients participating in the C-seal trial [3], we saw a similar clinical presentation. The C-seal is a biodegradable intraluminal device, which is attached proximal to a stapled colorectal anastomosis with the aim to reduce anastomotic leakage (Fig. 1(A)). In this short communication, we present three patients with a colon perforation proximal to the anastomosis, similar to a stercoral perforation, following a sigmoid resection or low anterior resection with application of a C-seal.

## Cases

We present three cases, two male and one female, with an age between 67 and 78 years old, all with a medical history known for constipation. They underwent colorectal cancer resection with a stapled anastomosis with attachment the C-seal. In none of the patients, a defunctioning stoma was created and all bowels were filled with stool. A few days postoperative, the patients developed signs of peritonitis like fever, abdominal pain, and elevated infection parameters (CRP > 260 mg/L and leukocytes up to 15.6 × 10^9^/L). Additionally, radiological imaging showed free intraperitoneal air and intra-abdominal fluid after which all patients underwent a relaparotomy. The anastomoses were all intact, without signs of ischemia and with intact C-seals in situ, but all with bowel perforations proximal of the anastomoses. One of the patients had several other relaparotomies, but recovered. Another patient was readmitted and the third died after several readmissions, due to progression of disease.

## Discussion

The presented cases showed a similar postoperative course after a colorectal resection with primary stapled colorectal anastomosis and application of an intraluminal device (C-seal). The intraoperative findings are similar to findings described in the literature in patients with a stercoral perforation.

Stercoral perforation arises due to hard fecal mass and fecal impaction, leading to bowel distension, raised intraluminal pressure with a subsequent bowel perforation [1]. There are several factors associated with the formation of fecal impaction. A medical history of constipation is common [1, 4]. Therefore, stercoral perforation is usually seen in elderly, bed-ridden, or neuropsychiatric patients. Constipation leading to fecal impaction can also be a result of colonic hypomobility, inadequate dietary fiber, and low water intake [4]. Several drugs, as narcotics, opioids, antidepressants, and aluminum-based antacids, are associated with constipation and fecal impaction as well [5]. Even the use of non-steroidal anti-inflammatory drugs is reported as a causative factor in the literature [6]. In addition, congenital and acquired conditions of the colon and rectum, such as megacolon, Hirschsprung disease, adhesive obstructions, and anatomic and functional abnormalities, have been mentioned as causes for fecal impaction [4]. Furthermore, colorectal cancer is associated with constipation, especially in patients with a stenosing tumor, narrowing the lumen of the colon and leading to proximal fecal impaction, which could lead to fecal impaction [7]. The patients in our cases all had a medical history showing constipation and two patients used medication that are known to cause constipation. Besides, during and after surgery, opioids were used for the pain management in all these cases.

Change of functional outcome after colorectal resection with an anastomosis, ranging from fecal incontinence to constipation, is described in the literature. Dysmotility of the colon after gastro-intestinal surgery is a known phenomenon and associated with less electrical activity in the colon. Furthermore, the innervation of the last part of the colon is mostly ligated during colorectal resection. This denervation often results in defecatory disorders, decreased colonic motility, prolonged colonic transit time and high intraluminal pressures in the denervated part [8].

Other studies are contradictory and show that denervation could result in increased motility of the distal colon [9]. The increased motility could cause propulsion of fecal mass [10]. These mass movements of the descending colon will push the fecal content through the anastomosis. In patients with a C-seal attached to the anastomosis, the fecal contents might congest in the C-seal while the content is distally propulsed. These mass movements cause high intraluminal pressure proximal to the anastomosis. When bowel contents are not pushed through the C-seal, possibly due to a dry environment and the absence of peristalsis in the C-seal, bowel contents subsequently accumulate proximal to the anastomosis. This fecal accumulation causes traction on the bowel wall proximal to the anastomosis and on the anastomosis itself. Consequently, this could lead to a blowout in the already vulnerable proximal bowel wall, causing (stercoral) perforation.

The most common site of fecal impaction is the sigmoid and rectosigmoid, because fecal content is maximally dehydrated in this part of the colon after resorption in the more proximal colon [1, 2]. Both the narrow diameter and the accumulation of feces result in a high intraluminal pressure in the distal part of the colon. The high intraluminal pressure might compromise capillary vessels, resulting in a reduced perfusion of the colon. The decreased perfusion makes the bowel wall more vulnerable for perforation [2]. This mechanism is also found in stercoral perforation, where the antimesenteric site, because of its relatively poor blood supply, is the most frequent site of perforation [1]. During surgical resection of the sigmoid or rectum surrounding arteries and veins are ligated, resulting in a compromised blood flow at the site of the anastomosis [11], which could contribute to the previously mentioned dysmotility of the colon, making the colonic wall more vulnerable at this site.

A colorectal anastomosis with a C-seal applied has a slightly narrower lumen than the rest of the colon and forms a dryer surrounding than the normal bowel mucosa; furthermore, there is a lack of motility, with less to no bowel movements distal to the anastomosis because of the C-seal. Due to this combination, it is comprehensible that dry and hardened fecal mass does not pass through the C-seal. Moreover, when in patients with an intraoperative full colon, as seen in patients who did not receive oral mechanical bowel preparation, a primary anastomosis is made with a C-seal (Fig. 1(B1)), it is plausible that feces accumulates proximal to the anastomosis causing obstruction. The fecal accumulation results in higher intraluminal pressure and may lead to pressure ulceration or necrosis (Fig. 1(B2)), with a subsequent bowel perforation proximal to the anastomosis (Fig. 1(B3)). A study with rats treated with an intraluminal device also showed obstruction in the bowel above a device [12].

The adverse postoperative outcome in our patients emphasizes the importance of a careful operative policy in patients with a medical history known for constipation undergoing a colorectal resection. It might be advisable to create a defunctioning stoma and, in some cases, it may even be better to decide not to make an anastomosis at all. At least, these patients should receive oral mechanical bowel preparation preoperatively, or when this is not possible because of a stenosing tumor process, preoperatively a defunctioning stoma could be constructed to reduce the risk of fecal impaction and its subsequent colonic perforation. Also in patients with an intraoperative colon full of luminal content, these considerations should be kept in mind. Because of these cases, a change was made in the protocol of the C-seal trial, after which patients had to be given oral mechanical bowel preparation before surgery in order to have an unfilled intestinal lumen or else they would be excluded. [3] After this protocol change, we did not see any perforations proximal to the anastomosis.

## Conclusion

Stercoral perforation of the colon is a rare phenomenon, for which no definitive treatment recommendations exist. Three patients treated with an intraluminal device after low anterior resection with a primary anastomosis showed a postoperative course similar to the clinical presentation of patients with stercoral perforation. This finding emphasizes the importance of careful surgical decision-making in anastomosis and stoma construction in patients with constipation, especially in patients treated with an intraluminal device.
